# Bridging Knowledge Gaps in Understanding SARS‐CoV‐2 Infection During Pregnancy

**DOI:** 10.1111/ppe.70050

**Published:** 2025-07-05

**Authors:** Anne K. Örtqvist, Maria C. Magnus, Stine Kjaer Urhoj

**Affiliations:** ^1^ Clinical Epidemiology Division, Department of Medicine, Solna Karolinska Institutet Stockholm Sweden; ^2^ Department of Obstetrics and Gynecology Visby Lasarett Visby Sweden; ^3^ Centre for Fertility and Health, Norwegian Institute of Public Health Oslo Norway; ^4^ Section of Epidemiology, Department of Public Health University of Copenhagen Copenhagen Denmark; ^5^ Statistics Denmark Copenhagen Denmark

Pregnant individuals have a comparable risk of contracting the severe acute respiratory syndrome coronavirus 2 (SARS‐CoV‐2) virus as nonpregnant individuals [1]. However, pregnancy increases the risk of developing severe COVID‐19, as defined by admission to the intensive care unit (ICU) or undergoing invasive ventilation, and a higher likelihood of death due to the disease [2].

This issue of *Paediatric and Perinatal Epidemiology* includes a study by Diguisto et al. [3], which aimed to advance our understanding of severe COVID‐19 by using a clinically nuanced definition of severe COVID‐19 based on organ dysfunction. This definition was applied through meticulous review of medical charts from a population‐based obstetric cohort spanning the first three waves of the pandemic [3]. We commend the authors for their rigorous efforts and for prioritising clinical relevance in defining disease severity. Based on this definition of severity, severe COVID‐19 affected one to nearly four per 1000 deliveries and 4%–9% of infected pregnant women across successive pandemic waves. Its prevalence was associated with risk factors, such as older age, migrant status, crowded living conditions, high BMI, chronic comorbidities, and infection occurring later in pregnancy, with neonatal complications primarily linked to severe maternal cases and often driven by medically induced prematurity [3].

In this commentary, we highlight remaining critical knowledge gaps regarding severe COVID‐19 in pregnancy, which must be addressed to enhance preparedness for future pandemics. These include the need for standardised definitions, tailored treatment strategies, inclusion of pregnant individuals in clinical trials, better understanding of maternal–fetal pathophysiology, long‐term follow‐up and ensuring that all countries—especially low‐ and middle‐income nations—have fair and adequate access to the tools, systems and resources needed to collect, analyse and share health data effectively during pandemics.

## 1 Knowledge Gaps

### 1.1 Definition of Severe COVID‐19

A major challenge in synthesising the evidence on severe COVID‐19 in pregnancy lies in the inconsistent definitions of severity across studies. There is currently no universally accepted or standardised set of criteria, resulting in heterogeneous case classifications that hinder cross‐country comparisons, including meta‐analyses. Although organ dysfunction‐based definitions, like the one used by Diguisto et al., offer a clinically grounded approach, they may reduce misclassification and allow for more consistent comparisons across populations and time. However, their universal adoption is far from reality. Moreover, such data requires manual chart review, which is resource‐intensive and not always feasible, especially in large‐scale or real‐time surveillance efforts.

Previous studies investigating the severity of COVID‐19 in pregnancy have predominantly relied on hospital or ICU admissions as proxies for severe disease [4]. This approach, however, presents limitations. Indications for hospital or ICU admission can vary widely across healthcare settings, countries and time periods, influenced by evolving knowledge of the disease, fluctuating caseloads and local resource availability. As a result, these proxies may introduce bias and limit comparability, potentially misclassifying disease severity. Additionally, they may not adequately reflect the unique physiological changes and vulnerabilities of pregnancy, nor capture the full spectrum of organ dysfunction or systemic complications associated with severe COVID‐19.

A core set of outcomes for maternal and neonatal health research and surveillance has been proposed for emerging and ongoing epidemic threats, in order to improve data comparisons and enable timely decision‐making [5]. However, a global consensus on severity criteria adapted for pregnancy‐specific physiology would enable meaningful international comparisons, support global health equity and improve the quality of research and clinical decision‐making. Future efforts should also explore how to extract these criteria automatically from electronic health records, using algorithms that integrate clinical, laboratory and imaging findings.

### 1.2 Distinguishing Maternal and Neonatal Risk

It is essential to recognise that severe maternal COVID‐19 does not necessarily equate to poor neonatal outcomes and vice versa. Pregnant individuals are more susceptible to severe infections due to a combination of physiological, mechanical and immunological changes that support fetal tolerance but may impair maternal antiviral responses [6]. Altered cytokine profiles, impaired interferon responses and changes in placental immunoregulation have been described among pregnant individuals but require further study [6]. Nonetheless, when assessing the relationship between severe maternal COVID‐19 and neonatal outcomes, clinical decision‐making plays a crucial role.

As Diguisto et al. have shown, severe maternal COVID‐19 often leads to preterm delivery, frequently via caesarean delivery [3]. Yet, studies report that even in the presence of maternal multi‐organ dysfunction, fetal Doppler assessments and placental transcriptomics may remain largely unaffected, suggesting preserved placental function also in the presence of severe maternal disease [7]. This issue prompts several critical considerations. Adverse neonatal outcomes may stem primarily from the pathophysiological effects of the virus or disease itself, or alternatively, from clinical interventions undertaken to safeguard maternal health. When clinical decisions are made chiefly in response to maternal distress, there may be consequential trade‐offs that affect neonatal well‐being. The indication for caesarean delivery must be carefully evaluated to distinguish between cases where it is medically necessary and those where it may be avoidable. Such decisions have the potential to significantly impact not only immediate neonatal outcomes but also maternal recovery trajectories and the health of future pregnancies. Without high‐quality evidence, clinical management often defaults to local protocols and expert opinion, underscoring the need for coordinated research and international guidelines.

### 1.3 Gaps in Clinical Management, Therapeutics, and Vaccination Trials

To date, there is limited evidence guiding the treatment of pregnant individuals with severe COVID‐19 [8]. Many therapies initially tested in the general population lack pregnancy‐specific data. This gap could lead to treatment hesitancy, delayed interventions, or the empirical use of therapies without robust safety or efficacy data in pregnancy. The introduction of COVID‐19 vaccines led to a marked reduction in severe cases, including among pregnant individuals. However, early vaccine trials excluded pregnant participants, delaying access and contributing to initial vaccine hesitancy. It has been estimated that the inclusion of pregnant women in trials and equitable post‐trial vaccine uptake could have prevented a substantial proportion of maternal deaths and stillbirths during the pandemic [9]. These experiences underscore the need to investigate the possibility of including pregnant individuals in clinical trials, while ensuring robust pharmacovigilance systems are in place.

### 1.4 Long‐Term Consequences: Maternal and Infant Health

Another critical gap lies in the long‐term follow‐up of both mothers and infants affected by severe COVID‐19. Data on post‐acute sequelae, such as long COVID, persistent cardiopulmonary symptoms and mental health impacts, remain scarce in the pregnant population. Similarly, little is so far known about the long‐term neurodevelopmental or health outcomes of infants born to mothers with severe COVID‐19, particularly those delivered preterm or exposed in utero to severe maternal inflammation or hypoxia. Future studies must prioritise longitudinal designs, include appropriate control groups and assess outcomes beyond the perinatal period, ideally spanning early childhood and subsequent pregnancies.

### 1.5 Future Surveillance Strategies

There is a growing body of evidence that differences in the variants of the SARS‐CoV‐2 virus can significantly influence maternal outcomes [4]. As new variants emerge, they may differ in transmissibility, virulence and impact on specific populations, including pregnant individuals. This variability reinforces the need for adaptive clinical guidelines and ongoing genomic surveillance. Health systems must be prepared to adjust protocols, resource allocation and communication strategies in response to evolving conditions and knowledge.

The manual review of medical records, as done in Diguisto et al., while thorough, is inherently slow, labour‐and resource‐intensive, and often impractical at scale. Population‐based registry data, when well‐integrated and linked in real time, offer a powerful alternative. In the Scandinavian countries, national electronic health records and data linkage across clinical, laboratory and public health systems have enabled near real‐time surveillance of COVID‐19 in pregnancy. This model has facilitated rapid case identification, assessment of vaccine safety and comparison across countries with differing testing policies and lockdown strategies [1, 4, 10].

However, this infrastructure is not universally available. Many countries lack integrated perinatal data systems, and low‐ and middle‐income countries are often underrepresented in the literature. This limits the generalisability of findings and hampers global preparedness. As Diguisto et al. emphasise, the creation of a permanent, international perinatal surveillance system is imperative. Such systems would not only enhance our ability to respond to future pandemics but also improve routine monitoring of maternal and neonatal health, enabling earlier detection of emerging threats and disparities. Rather than relying on ad hoc research funding or the dedication of individual investigators, these systems must be institutionally and politically prioritised to ensure sustained impact.

## 2 Conclusions

Diguisto et al. provide valuable insights into severe COVID‐19 in pregnancy by adopting a clinically grounded definition of severity based on organ dysfunction. Their findings highlight the importance of moving beyond administrative proxies and underscore the urgent need for consistent definitions, targeted research, equitable data infrastructure and global collaboration. Differences in testing strategies, severity definitions and healthcare systems have made it difficult to compare findings across countries. These inconsistencies have hindered the development of global guidelines and limited our understanding of the pandemic's true impact on maternal and perinatal health. International collaboration to harmonise definitions, surveillance methods and reporting standards would enhance our collective ability to respond to future pandemics with coordinated, evidence‐based strategies. As we reflect on the lessons of this pandemic, it is vital to address the many remaining knowledge gaps in maternal COVID‐19 care. Strengthening surveillance systems, investing in inclusive research and ensuring that pregnant individuals are not left behind in future public health responses are essential steps towards better maternal and perinatal outcomes—now and in the future.

## Author Contributions

A.K.Ö. drafted the original manuscript. M.C.M. and S.K.U. critically reviewed and revised the draft. All authors are part of the SCOPE collaboration (Scandinavian studies of COVID‐19 in Pregnancy).

## Conflicts of Interest

The authors declare no conflicts of interest.

## Data Availability

The authors have nothing to report.
